# Uncovering the genetic architecture of *Colletotrichum lindemuthianum* resistance through QTL mapping and epistatic interaction analysis in common bean

**DOI:** 10.3389/fpls.2015.00141

**Published:** 2015-03-17

**Authors:** Ana M. González, Fernando J. Yuste-Lisbona, A. Paula Rodiño, Antonio M. De Ron, Carmen Capel, Manuel García-Alcázar, Rafael Lozano, Marta Santalla

**Affiliations:** ^1^Grupo de Biología de Agrosistemas, Misión Biológica de Galicia-CSICPontevedra, Spain; ^2^Departamento de Biología y Geología (Genética), Centro de Investigación en Biotecnología Agroalimentaria (BITAL), Universidad de AlmeríaAlmería, Spain

**Keywords:** anthracnose, epistasis, *Phaseolus vulgaris* L., QTL, resistance gene clusters, NL genes

## Abstract

*Colletotrichum lindemuthianum* is a hemibiotrophic fungal pathogen that causes anthracnose disease in common bean. Despite the genetics of anthracnose resistance has been studied for a long time, few quantitative trait loci (QTLs) studies have been conducted on this species. The present work examines the genetic basis of quantitative resistance to races 23 and 1545 of *C. lindemuthianum* in different organs (stem, leaf and petiole). A population of 185 recombinant inbred lines (RIL) derived from the cross PMB0225 × PHA1037 was evaluated for anthracnose resistance under natural and artificial photoperiod growth conditions. Using multi-environment QTL mapping approach, 10 and 16 main effect QTLs were identified for resistance to anthracnose races 23 and 1545, respectively. The homologous genomic regions corresponding to 17 of the 26 main effect QTLs detected were positive for the presence of resistance-associated gene cluster encoding nucleotide-binding and leucine-rich repeat (NL) proteins. Among them, it is worth noting that the main effect QTLs detected on linkage group 05 for resistance to race 1545 in stem, petiole and leaf were located within a 1.2 Mb region. The NL gene Phvul.005G117900 is located in this region, which can be considered an important candidate gene for the non-organ-specific QTL identified here. Furthermore, a total of 39 epistatic QTL (E-QTLs) (21 for resistance to race 23 and 18 for resistance to race 1545) involved in 20 epistatic interactions (eleven and nine interactions for resistance to races 23 and 1545, respectively) were identified. None of the main and epistatic QTLs detected displayed significant environment interaction effects. The present research provides essential information not only for the better understanding of the plant-pathogen interaction but also for the application of genomic assisted breeding for anthracnose resistance improvement in common bean through application of marker-assisted selection (MAS).

## Introduction

Members of the ascomycete genus *Colletotrichum* cause devastating anthracnose diseases in many agronomically important crops in temperate, tropical and subtropical regions (Bailey and Jeger, 1992). The specialized hemibiotrophic fungus *C. lindemuthianum* [(Sacc. & Magnus) Lams. - Scrib]. has a reduced number of plant hosts, mainly common bean (*Phaseolus vulgaris* L.), although in less extent and severity, it can also colonize *P. acutifolius* var. *lactifolius*, *P. coccineus*, *P. aureus*, *P. lunatus*, *P. limensis*, *Medicago sativa*, and *Vicia faba* (Sicard et al., 1997; Mahuku et al., 2002). The pathogen has a sequential biotrophic- and necrotrophic-infection process to invade and colonize the plant hosts, that involves the transition from an asymptomatic biotrophic phase (characterized by intracellular thick primary hyphae) to a destructive necrotrophic phase (characterized by thin filamentous secondary hyphae) referred to as the biotrophynecrotrophy switch, which is essential for anthracnose disease development (Bhadauria et al., 2011). The remarkable resistance of *C. lindemuthianum* and its capacity for survivability in any environmental condition renders its presence responsible for losses in crops. In fact, the damage caused by this fungus in bean crops is so great that it has produced an economical loss in productive countries (Vigidal-Filho et al., 2007). Besides, *C. lindemuthianum* causes a hypersensitive response in bean resistant plants - groups of red-brownish wounds of different sizes that are produced by the plant to delimit the spread of the pathogenic fungus (Martínez-Pacheco et al., 2009). The process of co-evolution between the fungus and bean resistant plants has led this fungal species to produce new pathogenic variants, which can be detected on the basis of the phenotypic response to anthracnose infection shown by different varieties of common bean (Melotto et al., 2000; Rodríguez-Guerra et al., 2003). Thus, more than 100 races have been described for *C. lindemuthianum* (Rodríguez-Guerra et al., 2003) and new pathotypes are reported every day, indicating a large pathogenic variability of this fungus.

In common bean, up to 40 genes conferring resistance to specific races (designated as *Co*-) have been described, mainly due to *C. lindemuthianum* pathogenic variability. Anthracnose resistance is related to the presence of closely linked race-specific loci, which comprise different single, duplicate or complementary dominant genes, except for the recessive *co-8* gene (Kelly and Vallejo, 2004; Ferreira et al., 2013; Campa et al., 2014). Based on the hypothesis that the same gene confers the resistance to different races in a bean genotype, most classical studies considered that different resistance spectra in genotypes were due to different alleles of the same gene. As a result, different alleles were described for genes *Co-1*, *Co-3*, and *Co-4* (Kelly and Vallejo, 2004; Ferreira et al., 2013). Most identified resistance genes have been mapped on the different linkage groups (LG) of the common bean genetic map: genes *Co-1*, *Co-1*^*2*^, *Co-1*^*3*^, *Co-1*^*4*^, *Co-1*^*5*^, *Co-1*^*65-X*^, *Co-1*^*73-X*^, *Co-x*, and *Co-w* were mapped on LG01 (Barrus, 1915; Melotto and Kelly, 2000; Méndez-Vigo, 2001; Gonçalves-Vidigal and Kelly, 2006; Rodríguez-Suárez et al., 2007; Geffroy et al., 2008; Vallejo and Kelly, 2008; Gonçalves-Vidigal et al., 2011; Campa et al., 2014); *CoPv02c*^*3-X*^, *CoPv02c*^*7-X*^, *CoPv02c*^*19-X*^, *CoPv0c2*^*449-X*^, and *Co-u* on LG02 (Kelly et al., 2003; Campa et al., 2014); *Co-1*^*3*^ on LG03 (Lacanallo et al., 2010); *Co-3*, *Co-3c*^*3-X*^, *Co-3c*^*7-X*^, *Co-3c*^*19-X*^, *Co-3c*^*449-X*^, *Co3c*^*453-X*^, *Co-9*, *Co-y*, *Co-z*, *Co-10*, and *Co-15* on LG04 (Geffroy et al., 1999; Alzate-Marín et al., 2003; Méndez-Vigo et al., 2005; Rodríguez-Suárez et al., 2007, 2008; Gonçalves-Vidigal et al., 2013; Sousa et al., 2013; Campa et al., 2014); *Co-5*, *Co-6*, and *Co-v* on LG07 (Fouilloux, 1976; Campa et al., 2009); *Co-4* on LG08 (Melotto et al., 2004; Rodríguez-Suárez et al., 2007; Campa et al., 2014); *CoPv09c*^*453-C*^ on LG09 (Campa et al., 2014); and *Co-2*, *Co-2*^*6-C*^, *Co-2*^*39-C*^, *Co-2*^*38-C*^, *Co-2*^*357-C*^, *Co-2*^*65-C*^, *Co-2*^*7-C*^, *Co-2*^*3-C*^, *Co-2*^*19-C*^, *Co-2*^*449-C*^, and *Co-2*^*453-C*^ on LG11 (Adam-Blondon et al., 1994; Campa et al., 2014). Although genetic analyses support that the *Co*-genes behave as major Mendelian factors, they most likely exist as resistance gene clusters in which individual gene(s) confers resistance to one specific race. Most of the resistance-associated genes encode nucleotide-binding and leucine-rich repeat proteins, which are known as NB-LRR (NL) genes (Meyers et al., 2005). The presence of these clusters is widespread among higher plants, and clusters of NL genes have also been described in the common bean genome (Schmutz et al., 2014). In particular, two large clusters identified on chromosomes 4 and 11 could co-localize with previously mapped *Co-3* and *Co-2* anthracnose resistance genes, respectively.

Resistance to anthracnose in common bean generally follows a qualitative mode of inheritance where resistant and susceptible reactions are clearly differentiated. The specific resistance genes follow the classic gene-for-gene model (Flor, 1955), and the qualitative resistance provided by them is often less durable than quantitative resistance, since pathogens can more easily adapt to single gene-mediated resistance (St. Clair, 2010). In contrast, quantitative resistance usually confers broad-spectrum protection toward different races of biotrophic or necrotrophic pathogens (Oliver and Ipcho, 2004). The genetic regulation of quantitative traits is often complex due to their polygenic nature. However, trait dissection through Quantitative Trait Loci (QTL) analysis is a useful approach to identify chromosomal regions harboring genes that control these quantitative traits. Yet, in addition to mapping main effect QTLs, epistatic interactions between QTLs are important. Identification of quantitative disease resistance main and epistatic effects from multiple environments does not only help to extend the applicability of results, but is also essential for the development of an efficient marker-assisted selection (MAS) program aimed at improving breeding efficiency.

Despite the fact that genetics of anthracnose resistance in common bean has been studied for a long time, few QTL studies have been conducted on this species. The present work studies the genetic basis of quantitative resistance to two races of *C. lindemuthianum* in different organs of a segregating common bean recombinant inbred line population (RIL) from the cross PMB0225 × PHA1037. Using multi-environment QTL mapping approach, race specific anthracnose resistance QTLs were identified showing significant main additive effects in stem, petiole and leaf organs, which were co-localized with NL genes. In addition to identifying main effect QTLs, this analysis revealed epistatic interactions that explained phenotypic variation beyond those controlled by main effects of individual loci. Thus, markers associated with QTLs reported here constitute useful tools for MAS breeding programs directed toward improved anthracnose resistance.

## Materials and methods

### Biological material

A RIL population consisting of 185 F_7_ lines was developed by single-seed descent from an F_2_ population from the cross between PMB0225 (a common bean line as P1) and PHA1037 (nuña bean line abbreviated as P2) accessions belonging to the Andean gene pool. Mesoamerican (17, 73, 448, and 1545) and Andean (7, 23, 39, 55, and 102) anthracnose races were inoculated to the two parents. The twelve differential cultivars (Michelite, MDRK, Perry Marrow, Cornell 49242, Widusa, Kaboon, Mexico 222, PI207262, TO, TU, AB136, and G2333) were used to confirm the identity of the *C. lindemuthianum* races. Only races 23 and 1545 were pathogenic on PMB0225 parent and chosen for the present study.

### Plant growth conditions, inoculation, and disease evaluation

Plants were grown in plastic pots containing a mixture of clay soil and organic compound (1:1; v/v), under natural and artificial (12-h photoperiod, 166 μE s^−1^ m^−2^) photoperiod growth conditions with average day and night temperatures of 25 and 20°C, respectively. Plants were irrigated according to water needs. The anthracnose races were kept on potato-dextrose agar (PDA) at 19–21°C in darkness. To obtain conidia, fungus was grown for sporulation for about 15 days, and medium plates were flooded with 10 mL of 0.01% Tween 80 in distilled water. The conidial suspension was collected and filtered twice to remove mycelial fragments. The number of conidia was estimated using a haemocytometer and inoculum concentration was adjusted to 3 × 10^6^ conidia mL^−1^ with distilled water. Spore suspension was sprayed-inoculated onto 2-week-old bean plants showing the fully expanded primary leaves using an atomizer. Inoculated plants were sealed in order to increase 95–100% humidity for 48 h.

**Figure 1:**
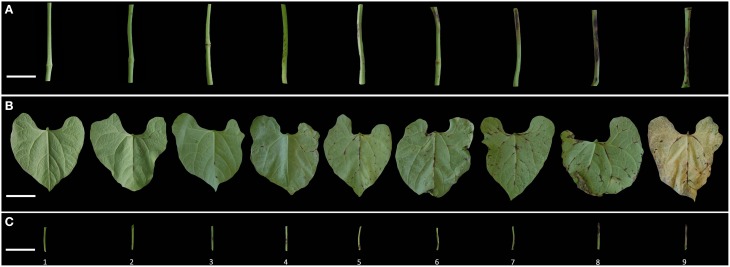
**Anthracnose infection phenotypes in stem (A), leaf (B), and petiole (C)**. Scale bar 1 cm.

The infected phenotypes were assessed on the basis of symptom severity on the primary leaves (L), stems (S), and petioles (P) at intervals of 7, 14, and 21 days post-inoculation (dpi). Numerical disease scores (DC) were assigned based on visual appreciation of the percentage of the organ presenting symptoms. A score of 1 represented no observed symptoms, while 9 corresponded to 100% of the organ covered by brown typical lesions of anthracnose (Figure 1). The Area Under the Disease Progress Curve (AUDPC) was calculated according to Shaner and Finney (1977) as: AUDPC = $\sum_{\text{i }=\;1}^{\text{n}}[{\text{x}}_{\text{i}}+{\text{x}}_{i\;+\;1}/2]\text{ t}$, where x_i_ is the disease score on date i, n the number of evaluations made and the time in days between evaluations x_i_and x_i + 1_. The use of AUDPC is an effective method to take both duration and severity of disease into account.

### Experimental design and statistical data analysis

The experiment was set up as a randomized complete block design with four replicates in artificial (named A-Ppd) and natural (named N-Ppd) photoperiod conditions, respectively. Each RIL genotype was represented by one plant in each block. Independent four-block experiments were carried out for each race, and the parental lines PMB0225 and PHA1037 were included.

Descriptive statistical parameters (mean value, standard deviation and range of variation) and normality (Kolmogorov–Smirnov test) were obtained for each quantitative trait and environment. Variation in the expression of traits through the environments was analyzed using PROC MIXED (SAS Institute Inc V. 9.04, Cary, NC, USA). Variance components and broad-sense heritabilities with their standard errors were estimated by restricted maximum likelihood (REML) option of the PROC MIXED and IML (SAS Institute Inc. v. 9.04, Cary, NC, USA) for the phenotypic traits (Holland et al., 2003; Holland, 2006). Phenotypic Pearson correlation coefficients among traits were implemented using PROC CORR across the environments (SAS Institute Inc. v. 9.04, Cary, NC, USA).

### QTL analysis

The genetic linkage map described by Yuste-Lisbona et al. (2012) was used for QTL analysis. The SCAR SW13 and SW12 (Fourie et al., 2004; Rodríguez-Suárez et al., 2008), 1 AFLP, 3 SSR, 29 SNP, and the seed coat color gene (*P*) were added to this map, which finally consisted of 229 loci (86 AFLP, 98 SSR, 42 SNP, 2 SCAR, and *P* locus) distributed on 11 LGs. The map spanned 858.4 cM, with an average distance of 3.7 cM between adjacent markers. Marker data were analyzed by JoinMap® 4.0 software (Van Ooijen, 2006). A minimum logarithm of odds ratio (LOD) score of 6.0 and a recombination frequency value of 0.3 were set as the linkage threshold for grouping markers. The Kosambi map function (Kosambi, 1944) was used to calculate the genetic distance between markers. The LGs were designated according to Pedrosa-Harand et al. (2008). QTLNetwork 2.0 software (Yang et al., 2008) was used to identify single-locus QTL, epistatic QTL (E-QTL) and their environment interaction effects (QTL × Environment, QE; and E-QTL × Environment, E-QE). The mixed-model based on composite interval mapping method (MCIM) was carried out for one-dimensional genome scan to detect putative single-locus QTL (defined as those showing significant main additive effects) and their environment interactions. In addition, a two-dimensional genome scan was carried out to identify epistatic interaction effects. An experimental-wise significance level of 0.05 was designated for candidate interval selection, putative QTL detection, and QTL effect. Both testing and filtration window size were set at 10 cM, with a walk speed of 1 cM. The critical *F-value* to declare putative QTLs was determined by a 1000 permutation test at 95% confidence level. The effects of QTL and environment interactions were estimated by the Markov Chain Monte Carlo method (Wang et al., 1994). QTL with only genetic effects indicated that these were expressed in the same way across environments. In addition, QTL with environment interaction effects suggested that their expressions were environmentally dependent. The detected QTLs were designated as recommended by Miklas and Porch (2010). The genetic map and the QTL detected were drawn using the MapChart 2.2 software (Voorrips, 2002).

### Identifying location of QTL in common bean genome

Nucleotide sequences of the markers flanking the main effect QTLs were used as queries for BLASTN search (Altschul et al., 1997) against the first chromosome scale version of *P. vulgaris* genome (Schmutz et al., 2014) available in the Phytozome database (http://www.phytozome.net/).

## Results

### Resistance variation in the RIL population

The bean accession PMB0225 was fully susceptible to anthracnose infection to race 1545 in all tested organs, and displayed susceptibility in leaf, intermediate resistance in stem, and full resistance in petiole to race 23. The PHA-1037 accession was fully resistant to both races in all organs. Table 1 shows the mean values and standard errors of the parental genotypes and the RIL population, as well as the ranges of variation of the RIL population for the resistance traits for each environment. In the RIL population, a continuous but bimodal distribution skewed toward the resistant parent PHA1037 was found regardless of the organ and race tested (Figure S1). The relative skewedness toward the resistant PHA1037 parent would imply that multiple genes with complementary additive effects are conferring resistance to anthracnose. Variance analysis was conducted for each environment and difference between blocks was not significant for most of the environments and resistant traits (Table 1). PMB0225 and PHA1037 parents and RIL progeny were significantly different for resistance traits in each environment (*P* = 0.001), demonstrating a genetic origin for the different levels of resistance in the RIL population.

**Table 1:** **Estimates of means, standard errors, range of variation, and variance analysis results for anthracnose resistance to races 23 and 1545 of the two common bean parents, PMB0225 and PHA1037, and the RIL population, grown in two environments (Env)**.

**Env**	**Block**	**Parents**			**RILs**			
		**PMB0225**	**PHA-1037**	**P^a^_PAR_**	**N^b^**	**Mean**	**Range**	**P_RIL_**
**SDC RACE 23**								
A-Ppd	ns	5.00 ± 0.00	1.00 ± 0.00	**	177	2.96 ± 0.10	1.00–9.00	**
N-Ppd	ns	5.50 ± 0.50	1.00 ± 0.00	**	177	2.42 ± 0.07	1.00–9.00	**
**SDC RACE 1545**								
A-Ppd	ns	9.00 ± 0.00	1.00 ± 0.00	**	178	2.92 ± 0.10	1.00–9.00	**
N-Ppd	ns	8.75 ± 0.25	1.00 ± 0.00	**	178	4.54 ± 0.11	1.00–9.00	**
**SAUDPC RACE 23**								
A-Ppd	ns	765.00 ± 13.00	156.00 ± 0.00	**	177	447.75 ± 15.93	156.56–1400.00	**
N-Ppd	ns	855.50 ± 77.50	156.00 ± 0.00	**	177	333.08 ± 10.31	156.56–1400.00	**
**SAUDPC RACE 1545**								
A-Ppd	ns	1400.00 ± 0.00	156.00 ± 0.00	**	178	367.49 ± 13.09	155.56–1400.00	**
N-Ppd	*	1244.25 ± 72.74	156.00 ± 0.00	**	178	688.24 ± 17.36	155.56–1400.00	**
**PDC RACE 1545**								
A-Ppd	*	9.00 ± 0.00	1.00 ± 0.00	**	178	2.73 ± 0.10	1.00–9.00	**
N-Ppd	ns	9.00 ± 0.00	1.00 ± 0.00	**	178	2.78 ± 0.12	1.00–9.00	**
**PAUDPC RACE 1545**								
A-Ppd	**	1400.00 ± 0.00	156.00 ± 0.00	**	178	367.55 ± 13.19	156.56–1400.00	**
N-Ppd	ns	1302.75 ± 73.58	156.00 ± 0.00	**	178	364.71 ± 15.93	156.56–1400.00	**
**LDC RACE 23**								
A-Ppd	ns	6.33 ± 0.33	1.00 ± 0.00	**	177	3.10 ± 0.11	1.00–9.00	**
N-Ppd	**	8.50 ± 0.50	1.00 ± 0.00	**	177	2.47 ± 0.08	1.00–9.00	**
**LDC race 1545**								
A-Ppd	**	9.00 ± 0.00	1.00 ± 0.00	**	178	3.66 ± 0.10	1.00–9.00	**
N-Ppd	ns	9.00 ± 0.00	1.00 ± 0.00	**	178	3.79 ± 0.13	1.00–9.00	**
**LAUDPC RACE 23**								
A-Ppd	ns	868.33 ± 84.92	168.67 ± 12.67	**	177	464.71 ± 17.06	156.56–1400.00	**
N-Ppd	**	1322.00 ± 78.00	156.00 ± 0.00	**	177	326.45 ± 9.79	156.56–1400.00	**
**LAUDPC RACE 1545**								
A-Ppd	**	1400.00 ± 0.00	156.00 ± 0.00	**	178	515.05 ± 15.17	156.56–1400.00	**
N-Ppd	ns	1302.75 ± 73.58	156.00 ± 0.00	**	178	560.00 ± 19.42	156.56–1400.00	**

a ns, not significant differences;

*, ** *significant at the 0.05 and 0.01 probability levels, respectively, for difference among parents (P_PAR_), RILs (P_RIL_), and block effect*.

b *N number of lines recorded*.

*SDC, stem disease score; SAUDPC, stem area under the disease progress curve; PDC, petiole disease score; PAUDPC, petiole area under the disease progress curve; LDC, leaf disease score; LAUDPC, leaf area under the disease progress curve*.

The estimated broad-sense heritability estimates for resistance traits between organs for a given race were high, with values ≥0.70 (Table 2). These values are in accordance with those reported by Geffroy et al. (2000) for anthracnose resistance between aerial organs of the plant to isolates 45 and A7, with heritability values ranging from 0.90 to 0.98. There was a strong correlation (*P* ≤ 0.001) for disease resistance scores between the different organs for a given race (Table 2). This was in sharp contrast to the absence of correlation when the data regarding both races were compared. These findings suggest that different genes could be involved in resistance for a given race, while identical genes condition resistance against the same race in different organs. In addition and in order to determine if the same set of genes encode resistance against both races, co-segregations for resistance to races 23 and 1545 were considered. Twenty-nine and thirty-four RILs were resistant to each race 23 and 1545, respectively, while evidence of co-segregation of disease response to both races was observed in 114 RILs (14 and 74 RILs were susceptible and resistant to both races in all organs, and 26 RILs presented resistance to both races but not in all organs). Accordingly, these results suggest that different genes determined specific resistance to races 23 and 1545.

**Table 2:** **Phenotypic correlation coefficients and heritability estimates with their standard errors among resistance traits for anthracnose races 23 and 1545**.

**Trait**	**Race 23**				**Race 1545**					***h*^2^ ± S.E.**
	**SDC**	**SAUDPC**	**LDC**	**LAUDPC**	**SDC**	**SAUDPC**	**PDC**	**PAUDPC**	**LDC**	
**RACE 23**										
SDC										0.87 ± 0.02
SAUDPC	0.97^**^									0.72 ± 0.04
LDC	0.85^**^	0.82^**^								0.91 ± 0.01
LAUDPC	0.81^**^	0.82^**^	0.97^**^							0.78 ± 0.03
**RACE 1545**										
SDC	0.07	0.03	0.02	−0.01						0.93 ± 0.01
SAUDPC	0.07	0.02	0.01	−0.03	0.96^**^					0.88 ± 0.02
PDC	0.13^*^	0.11	0.06	0.05	0.76^**^	0.72^**^				0.93 ± 0.01
PAUDPC	0.13^*^	0.11	0.07	0.06	0.70^**^	0.67^**^	0.95^**^			0.90 ± 0.01
LDC	0.14^*^	0.11	0.11	0.10	0.81^**^	0.77^**^	0.85^**^	0.79^**^		0.90 ± 0.02
LAUDPC	0.13^*^	0.10	0.10	0.08	0.82^**^	0.79^**^	0.85^**^	0.81^**^	0.99^**^	0.79 ± 0.03

*,** *Significant at the 0.05 and 0.01 probability levels, respectively*.

*SDC, stem disease score; SAUDPC, stem area under the disease progress curve; PDC, petiole disease score; PAUDPC, petiole area under the disease progress curve; LDC, leaf disease score; LAUDPC, leaf area under the disease progress curve*.

### Mapping of main effect QTL

**Figure 2:**
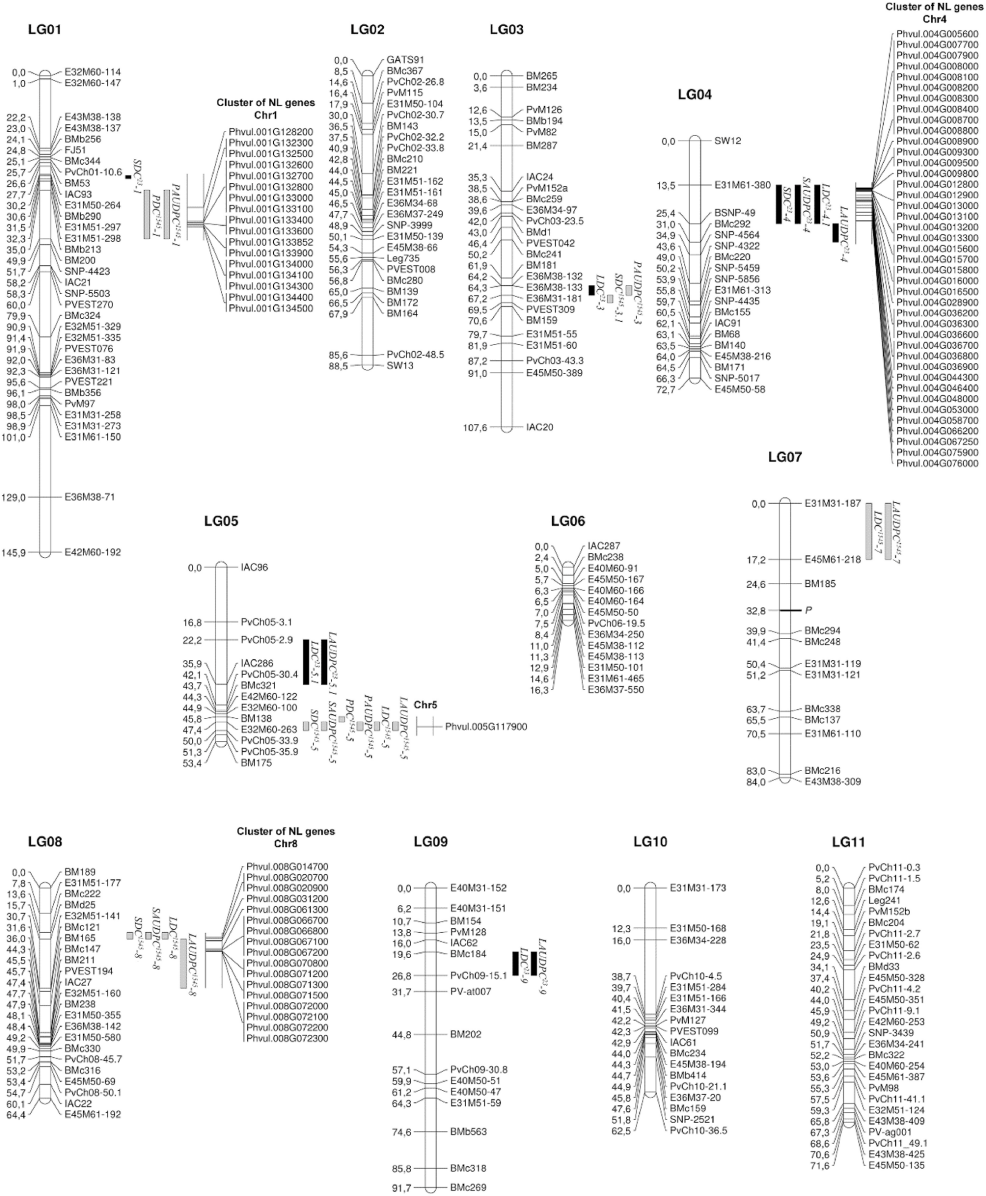
**Location of main effect QTLs for resistance to anthracnose races 23 and 1545 on a genetic linkage map of common bean based on the RIL population developed from the cross PMB0225 × PHA1037**. Distances among markers are indicated in cM to the left of the linkage groups (LG); names of markers are shown on the right. QTLs are depicted as vertical bars to the right of the LG. QTL detected for resistance to race 23 are indicated in black. QTLs identified for resistance to race 1545 are shown in gray. Co-location of resistance-associated genes encoding NB-LRR (NL) proteins with anthracnose QTLs are represented to the right of the QTLs.

The evaluation of the RIL population developed from the cross PMB0225 x PHA1037 under different environments has led to the identification of 10 and 16 main effect QTLs involved in resistance against anthracnose races 23 and 1545, respectively. These QTLs were mapped on eight LGs, with the exception of LGs 02, 10, and 11 (Figure 2). All QTLs detected had significant additive effects and did not display significant additive-by-environment interaction effects. A complete report of the single-locus QTL detected for anthracnose resistance traits is given in Table 3.

**Table 3:** **Single-locus QTL effects for resistance to anthracnose races 23 and 1545**.

**QTL**	**Marker interval**	**LG (pos.)^a^**	***F*-value^b^**	**A^c^**	***h*^2^(a)^d^**
**SDC—THRESHOLD *F*-VALUE: RACE 23 = 8.15, RACE 1545 = 8.05**					
*SDC*^*23*^-*1*	BMb290–E31M51-297	01 (30.57–31.52)	8.90	−0.67^***^	7.90
*SDC*^*23*^-*4*	E31M61-380–BSNP-49	04 (13.54–25.39)	9.92	−0.40^***^	3.03
*SDC*^*1545*^-*3.1*	E36M31-181–PVEST309	03 (67.19–69.54)	8.64	1.16^***^	4.97
*SDC*^*1545*^-*5*	E32M60-263–PvCh05-33.9	05 (47.37–49.99)	26.54	−0.94^***^	14.24
*SDC*^*1545*^-*8*	BMc222–BMd25	08 (13.63–15.71)	13.73	−0.70^***^	4.68
**SAUDPC—THRESHOLD *F*-VALUE: RACE 23 = 7.98, RACE 1545 = 8.26**					
*SAUDPC*^*23*^-*4*	E31M61-380–BSNP-49	04 (13.54–25.39)	9.17	−29.57^*^	2.64
*SAUDPC*^*1545*^-*5*	E32M60-263–PvCh05-33.9	05 (47.37–49.99)	25.92	−153.45^***^	13.32
*SAUDPC*^*1545*^-*8*	BMc222–BMd25	08 (13.63–15.71)	9.45	−87.49^***^	4.33
**PDC—THRESHOLD *F*-VALUE: RACE 1545 = 8.19**					
*PDC*^*1545*^-*1*	BMb213–BM200	01 (35.01–49.89)	9.98	1.39^***^	5.59
*PDC*^*1545*^-*5*	BM138–E32M60-263	05 (45.77–47.37)	18.49	−1.21^***^	10.22
**PAUDPC—THRESHOLD *F*-VALUE: RACE 1545 = 8.10**					
*PAUDPC*^*1545*^-*1*	BMb213–BM200	01 (35.01–49.89)	10.54	196.43^***^	5.03
*PAUDPC*^*1545*^-*3*	E36M38-133–E36M31-181	03 (64.29–67.19)	8.42	72.83^***^	4.08
*PAUDPC*^*1545*^-*5*	E32M60-263–PvCh05-33.9	05 (47.37–49.99)	15.73	−133.22^***^	8.28
**LDC—THRESHOLD *F*-VALUE: RACE 23 = 8.29, RACE 1545 = 8.16**					
*LDC*^*23*^-*3*	*E36M38-133–E36M31-181*	03 (64.29–67.19)	9.10	−0.57^***^	4.45
*LDC*^*23*^-*4.1*	E31M61-380–BSNP-49	04 (13.54–25.39)	9.73	−0.45^***^	3.30
*LDC*^*23*^-*5.1*	PvCh05-2.9–IAC286	05 (22.19–35.94)	14.07	−0.84^***^	5.98
*LDC*^*23*^-*9*	BMc184–PvCh09-15.1	09 (19.61–26.81)	14.93	−0.69^***^	11.43
*LDC*^*1545*^-*5*	E32M60-263–PvCh05-33.9	05 (47.37–49.99)	32.45	−1.71^**^	16.75
*LDC*^*1545*^-*7*	E31M31-187–E45M61-218	07 (0.00–17.21)	10.39	−0.46^**^	1.49
*LDC*^*1545*^-*8*	BMc222–BMd25	08 (13.63–15.71)	9.42	−0.54^**^	3.98
**LAUDPC—THRESHOLD *F*-VALUE: RACE 23 = 8.27, RACE 1545 = 8.07**					
*LAUDPC*^*23*^-*4*	BSNP-49–BMc292	04 (25.39–30.95)	8.90	−61.65^***^	3.06
*LAUDPC*^*23*^-*5.1*	PvCh05-2.9–IAC286	05 (22.19–35.94)	13.33	−84.09^***^	4.36
*LAUDPC*^*23*^-*9*	BMc184–PvCh09-15.1	09 (19.61–26.81)	14.72	−126.19^***^	10.19
*LAUDPC*^*1545*^-*5*	E32M60-263–PvCh05-33.9	05 (47.37–49.99)	33.39	−249.78^***^	16.75
*LAUDPC*^*1545*^-*7*	E31M31-187–E45M61-218	07 (0.00–17.21)	11.22	−68.43^***^	1.65
*LAUDPC*^*1545*^-*8*	BMd25–E32M51-141	08 (15.71–30.65)	8.33	−74.27^***^	3.80

a *Linkage group and the estimated confidence interval of QTL position in brackets (in Kosambi cM)*.

b *F-values of significance of each QTL*.

c *Estimated additive effect. Positive values indicate that alleles from PHA1037 have a positive effect on the traits, and negative values indicate that positive effect on the traits is due to the presence of the alleles from PMB0225*.

d *Percentage of the phenotypic variation explained by additive effects*.

*Experiment-wide P-value*.

* P = 0.05,

** P = 0.01,

*** *P = 0.001*.

*SDC, stem disease score; SAUDPC, stem area under the disease progress curve; PDC, petiole disease score; PAUDPC, petiole area under the disease progress curve; LDC, leaf disease score; LAUDPC, leaf area under the disease progress curve*.

Ten main effect QTLs were identified for resistance to race 23: one on each LGs 01 and 03, four on LG04, and two on each LGs 05 and 09 (Figure 2). Three of them had significant effects on stem resistance (SDC and SAUDPC traits), positioned on LGs 01 and 04, and explaining a phenotypic variance from 2.64 to 7.90%. The remaining QTLs were involved in leaf resistance (LDC and LAUDPC traits) on LGs 03, 04, 05, and 09, explaining a phenotypic variance from 3.06 to 11.43%. Two QTLs for resistance in leaf, *LDC*^*23*^-*9* and *LAUDPC*^*23*^-*9*, were co-localized on LG09, explaining 11.43 and 10.19% of the phenotypic variation, respectively. Likewise, the QTLs *LDC*^*23*^-*5.1* and *LAUDPC*^*23*^-*5.1* for resistance in leaf were co-localized on LG05, explaining 5.98 and 4.36% of the phenotypic variation, respectively. On LG04, four QTLs (*SDC*^*23*^-*4*, *SAUDPC*^*23*^-*4*, *LDC*^*23*^-*4.1*, and *LAUDPC*^*23*^-*4*) for resistance in stem and leaf were co-localized or nearly co-localized, indicating that this genomic region could confer a non-organ-specific resistance to anthracnose race 23. The total phenotypic variation explained by the main effect QTLs detected varied from 2.64 to 10.93% for stem traits (SAUDPC and SDC, respectively) and from 17.61 to 25.16% for leaf traits (LAUDPC and LDC, respectively). All these QTLs had negative additive values, which indicate that the increase in resistance is due to the presence of the alleles from PHA1037.

For resistance to race 1545, 16 main effect QTLs were found: two on each LG 01, 03, and 07, six on LG05, and four on LG08 (Figure 2). Five of them had significant effects on stem resistance (SDC and SAUDPC traits), positioned on LGs 03, 05, and 08, and explaining a phenotypic variance from 4.33 to 14.24%. For petiole resistance (PDC and PAUDPC traits), five QTLs were detected on LGs 01, 03, and 05, which explain a phenotypic variance from 4.08 to 10.22%. The remaining six QTLs were involved in leaf resistance (LDC and LAUDPC traits) on LGs 05, 07, and 08, with phenotypic variance explained ranging from 1.49 to 16.75%. Organ-specific QTLs were identified for petiole resistance on LG01 (*PDC*^*1545*^-*1* and *PAUDPC*^*1545*^-*1*) and for leaf resistance on LG07 (*LDC*^*1545*^-*7* and *LAUDPC*^*1545*^-*7*). Six main effect QTLs (*SDC*^*1545*^-*5*, *SAUDPC*^*1545*^-*5*, *PDC*^*1545*^-*5*, *PAUDPC*^*1545*^-*5*, *LDC*^*1545*^-*5*, and *LAUDPC*^*1545*^-*5*) were co-localized or nearly co-localized on LG05 for resistance to the three evaluated organs, showing the existence of non-organ-specific resistance to race 1545 in this genomic region. In addition, non-organ-specific QTLs were also found for stem and leaf on LG08 (*SDC*^*1545*^-*8*, *SAUDPC*^*1545*^-*8*, *LDC*^*1545*^-*8*, and *LAUDPC*^*1545*^-*8*), and for stem and petiole on LG03 (*SDC*^*1545*^-*3.1* and *PAUDPC*^*1545*^-*3*). The total phenotypic variation explained by the additive effects of all putative QTLs identified varied from 17.65 to 23.89% for stem traits (SAUDPC and SDC, respectively), from 15.81 to 17.39% for petiole traits (PDC and PAUDPC, respectively), and from 22.20 to 22.22% for leaf traits (LAUDPC and LDC, respectively). PHA1037 alleles were associated with resistance for most of the QTLs detected, with the exception of the QTLs *SDC*^*1545*^-*3.1, PDC*^*1545*^-*1*, *PAUDPC*^*1545*^-*1*, and *PAUDPC*^*1545*^-*3*, which had positive additive values, indicating that resistance alleles came from PMB0225.

The location of the anthracnose resistance main effect QTLs to races 23 and 1545 was different in most cases, which is in agreement with the absence of correlation between the resistance reactions against both races. Only the QTLs *LDC*^*23*^-*3*, *SDC*^*1545*^-*3.1*, and *PAUDPC*^*1545*^-*3* were co-localized or nearly co-localized on LG03 for resistance to race 23 in leaf, and to race 1545 in stem and petiole, although with opposite additive values (Table 3).

### Detection of epistatic QTL

A total of 39 E-QTLs (twenty-one for resistance to race 23 and 18 for resistance to race 1545) involved in 20 epistatic interactions (eleven and nine interactions for resistance to races 23 and 1545, respectively) were detected by the combined analysis of the multi-environment phenotypic values. None of the epistatic interactions detected displayed significant environment interaction effects. These E-QTLs were mapped on seven LGs, with the exception of LGs 02, 07, 10, and 11. The positive and negative additive-by-additive epistatic effect values obtained for these epistatic interactions indicate that both parent alleles could contribute to increasing the resistance to anthracnose races 23 and 1545. A complete description of digenic epistatic interaction analysis for anthracnose resistance traits toward both races is shown in Table 4.

**Table 4:** **Epistatic QTLs (E-QTLs) and E-QTL × Environment (E-QE) interaction effects for resistance to anthracnose races 23 and 1545**.

**E-QTLi^a^**	**Marker interval**	**LG (pos.)^b^**	**E-QTLj^a^**	**Marker interval**	**LG (pos.)**	***F*-value^c^**	**AA^d^**	***h*^2^(aa)^e^**
**SDC—THRESHOLD *F*-VALUE: RACE 23 = 8.28, RACE 1545 = 8.52**								
*E-SDC*^*23*^-*1*	BMb290–E31M51-297	01 (30.57–31.52)	*E-SDC*^*23*^-*4*	E31M61-380–BSNP-49	04 (13.54–25.39)	8.31	0.36^**^	2.65
*E-SDC*^*23*^-*3*	E36M31-181–PVEST309	03 (67.19–69.54)	*E-SDC*^*23*^-*5*	PvCh05-3.1–PvCh05–2.9	05 (16.80–22.19)	13.90	0.45^***^	4.20
*E-SDC*^*1545*^-*3.2*	PvCh03-23.5–BMd1	03 (42.04–42.99)	*E-SDC*^*1545*^-*9*	IAC62–BMc184	09 (15.99–19.61)	11.45	−0.83^***^	7.05
**SAUDPC—THRESHOLD *F*-VALUE: RACE 23 = 7.99, RACE 1545 = 7.49**								
*E-SAUDPC*^*23*^-*3.1*	IAC24–PvM152a	03 (35.29–38.48)	*E-SAUDPC*^*23*^-*3.2*	BM181–E36M38-132	03 (61.92–64.16)	10.38	199.14^***^	4.61
*E-SAUDPC*^*23*^-*3.3*	E36M38-133–E36M31-181	03 (64.29–67.19)	*E-SAUDPC*^*23*^-*5.1*	IAC96–PvCh05-3.1	05 (0.00–16.80)	14.38	85.30^***^	4.71
*E-SAUDPC*^*23*^-*5.2*	E32M60-100–BM138	05 (44.92–45.77)	*E-SAUDPC*^*23*^-*8*	BMc316–E45M50-69	08 (53.20–53.42)	10.15	−157.93^***^	6.74
*E-SAUDPC*^*1545*^-*1*	BM200–SNP-4423	01 (49.90–51.74)	*E-SAUDPC*^*1545*^-*6*	E40M60-166–E40M60-164	06 (6.35–6.53)	18.80	153.00^***^	6.38
**PDC—THRESHOLD *F*-VALUE: RACE 1545 = 6.27**								
*E-PDC*^*1545*^-*8*	BMc222–BMd25	08 (13.63–15.71)	*E-PDC*^*1545*^-*9*	E40M50-51–E40M50-47	09 (59.86–61.16)	7.41	−0.63^***^	3.34
**PAUDPC—THRESHOLD *F*-VALUE: RACE 1545 = 5.91**								
*E-PAUDPC*^*1545*^-*1*	BMb213–BM200	01 (35.01–49.89)	*E-PAUDPC*^*1545*^-*5*	E32M60-263–PvCh05-33.9	05 (47.37–49.99)	6.23	−174.10^***^	2.25
*E-PAUDPC*^*1545*^-*4*	SW12–E31M61-380	04 (0.00–13.54)	*E-PAUDPC*^*1545*^-*6*	E45M38-112–E45M38-113	06 (11.01–11.29)	11.41	98.29^***^	5.21
**LDC—THRESHOLD *F*-VALUE: RACE 23 = 8.17, RACE 1545 = 8.25**								
*E-LDC*^*23*^-*1.1*	IAC93–E31M50-264	01 (27.66–30.19)	*E-LDC*^*23*^-*3*	E36M38-133–E36M31-181	03 (64.29–67.19)	11.12	0.75^***^	7.92
*E-LDC*^*23*^-*1.2*	BMb290–E31M51-297	01 (30.57–31.52)	*E-LDC*^*23*^-*3*	E36M38-133–E36M31-181	03 (64.29–67.19)	10.24	−0.61^***^	2.94
*E-LDC*^*23*^-*5.2*	IAC286–PvCh05-30.4	05 (35.94–42.07)	*E-LDC*^*23*^-*8*	BMc316–E45M50-69	08 (53.20–53.42)	9.61	−1.03^***^	4.43
*E-LDC*^*1545*^-*3.1*	BMd1–PVEST042	03 (42.99–46.37)	*E-LDC*^*1545*^-*9*	BMc184–PvCh09-15.1	09 (19.61–26.81)	9.20	−0.64^***^	3.23
*E-LDC*^*1545*^-*3.2*	PVEST309–BM159	03 (69.54–70.61)	*E-LDC*^*1545*^-*3.3*	E45M50-389–IAC20	03 (91.01–107.61)	10.54	2.78^***^	6.66
**LAUDPC—THRESHOLD *F*-VALUE: RACE 23 = 7.81, RACE 1545 = 8.39**								
*E-LAUDPC*^*23*^-*4*	BSNP-49–BMc292	04 (25.39–30.95)	*E-LAUDPC*^*23*^-*5.1*	PvCh05-2.9–IAC286	05 (22.19–35.94)	8.12	62.67^**^	1.78
*E-LAUDPC*^*23*^-*5.1*	PvCh05-2.9–IAC286	05 (22.19–35.94)	*E-LAUDPC*^*23*^-*9*	BMc184–PvCh09-15.1	09 (19.61–26.81)	7.98	65.11^**^	1.13
*E-LAUDPC*^*23*^-*5.2*	E32M60-100–BM138	05 (44.92–45.77)	*E-LAUDPC*^*23*^-*8*	BMc316–E45M50-69	08 (53.19–53.42)	13.31	−145.89^***^	5.01
*E-LAUDPC*^*1545*^-*3.1*	BMd1–PVEST042	03 (42.99–46.37)	*E-LAUDPC*^*1545*^-*9*	BMc184–PvCh09-15.1	09 (19.61–26.81)	8.63	−91.19^***^	3.66
*E-LAUDPC*^*1545*^-*3.2*	PVEST309–BM159	03 (69.54–70.61)	*E-LAUDPC*^*1545*^-*3.3*	E45M50-389–IAC20	03 (97.01–107.61)	10.49	848.65^***^	5.79

a *E-QTLi and E-QTLj are the two QTLs involved in epistatic interaction*.

b *Linkage group and the estimated confidence interval of QTL position in brackets (in Kosambi cM)*.

c *F values of significance of each epistatic interaction*.

d *Estimated additive-by-additive epistatic effect. Positive values indicate that alleles from PHA1037 increase the trait value, and negative values indicate that the increase in the trait is due to the presence of the alleles from PMB0225*.

e *Percentage of the phenotypic variation explained by additive-by-additive epistatic effects*.

*Experiment-wide P-value*.

^*^P = 0.05,

** P = 0.01,

*** *P = 0.001*.

*SDC, stem disease score; SAUDPC, stem area under the disease progress curve; PDC, petiole disease score; PAUDPC, petiole area under the disease progress curve; LDC, leaf disease score; LAUDPC, leaf area under the disease progress curve*.

For resistance to race 23, 6 of the 21 E-QTLs identified were previously detected as main effect QTLs. Thus, not only did the E-QTLs *E-SDC*^*23*^-*1*, *E-SDC*^*23*^-*4*, *E-LDC*^*23*^-*3*, *E-LAUDPC*^*23*^-*4*, *E-LAUDPC*^*23*^-*5.1*, and *E-LAUDPC*^*23*^-*9* participate in epistatic interactions, but they also had an individual genetic effect. The analysis revealed novel loci on LGs 01, 03, 05, and 08 interacting so as to influence resistance to race 23. The percentage of phenotypic variance explained by the interaction of the E-QTLs ranged from 1.13 to 7.92%. Among the E-QTLs detected, it is noteworthy that the genomic region located between markers BMc316 and E45M50-69 on LG08 bears E-QTLs (*E-SAUDPC*^*23*^-*8*, *E-LDC*^*23*^-*8*, and *E-LAUDPC*^*23*^-*8*) involved in epistatic interactions for resistance in stem and leaf, which indicates that this region could participate in non-organ-specific resistance to anthracnose race 23. Collectively, the percentage of phenotypic variance explained by the interaction of the E-QTLs varied from 6.85 to 16.06% for stem traits (SDC and SAUDPC, respectively), and from 7.92 to 15.29% for leaf traits (LAUDPC and LDC, respectively).

Novel loci on LGs 01, 03, 04, 06, 08, and 09 were detected as involved in resistance to race 1545. Among the 18 E-QTLs identified, two E-QTLs were previously identified as main effect QTLs (*PAUDPC*^*1545*^-*1* and *PAUDPC*^*1545*^-*5*). The percentage of phenotypic variance explained by the interaction of the E-QTLs varied from 2.25 to 7.05%. All the E-QTLs detected for resistance to race 1545 were organ-specific. The total phenotypic variation explained by the additive-by-additive epistatic effects of all E-QTLs detected ranged from 6.38 to 7.05% for stem traits (SAUDPC and SDC, respectively), from 3.34 to 7.46% for petiole traits (PDC and PAUDPC, respectively), and from 9.45 to 9.89% for leaf traits (LAUDPC and LDC, respectively).

### Identifiying location of QTL in common bean genome

BLASTN analysis of the nucleotide sequences of the markers flanking the main effect QTLs with common bean genome revealed that the homologous regions spanning 17 of the 26 QTLs identified were positive for the presence of NL genes (Figure 2). Thus, the main effect QTLs *SDC*^*23*^-*1*, *PDC*^*1545*^-*1*, and *PAUDPC*^*1545*^-*1* covered 19.32 cM (30.57–49.89 cM) on LG01, while the corresponding genomic region covered 5.2 Mb on Chromosome (Chr) 1. Within this region, there is a cluster consisting of 17 NL genes. Likewise, the QTLs *SDC*^*23*^-*4*, *SAUDPC*^*23*^-*4*, *LDC*^*23*^-*4*, and *LAUDPC*^*23*^-*4* detected on LG04 (13.54–30.95 cM) were located within an important cluster of 41 NL genes on Chr4 (0.4–12.6 Mb). Meanwhile, the QTLs *SDC*^*1545*^-*5*, *SAUDPC*^*1545*^-*5*, *PDC*^*1545*^-*5*, *PAUDPC*^*1545*^-*5*, *LDC*^*1545*^-*5*, and *LAUDPC*^*1545*^-*5* covered 4.22 cM (45.77–49.99 cM) on LG05, whereas the homologous genomic regions spanned 1.2 Mb on Chr5 (32.7–33.9 Mb). The NL gene Phvul.005G117900 is located in this region, which encodes for a leucine-rich repeat (LRR) protein. In addition, the QTLs *SDC*^*1545*^-*8*, *SAUDPC*^*1545*^-*8*, *LDC*^*1545*^-*8*, and *LAUDPC*^*1545*^-*8* identified on LG08 (13.63–30.65 cM) were placed in a cluster of 17 NL genes on Chr8 (1.3–6.6 Mb).

## Discussion

To gain insight in basic knowledge dealing with resistance, a pathosystem that involves *C. Lindemuthianum* and *P. vulgaris* model legume has been characterized. The gene action governing anthracnose resistance was studied in a broad set of RILs generated from a cross between susceptible and resistant Andean accessions. Thus, insights into the number of quantitative resistance loci involved in anthracnose resistance to races 23 and 1545 were provided, as well as their epistatic interactions. The pathogen infected and colonized PMB0225 line, although symptoms and pathogen development were significantly reduced in race 23 as compared to race 1545. Genetic and molecular analysis revealed different features associated with the resistance of PHA1037.

### Genetic architecture of resistance

The phenotypic dissection of anthracnose resistance carried out in the RIL population has led to the detection of different kinds of resistance components. The pathogen resistance response was consistent between the testing environments, which evidenced that anthracnose resistance is mostly influenced by genes rather than environmental conditions. The occurrence of major resistance factors was found across both races and in the different organs tested. Moreover, genomic regions controlling anthracnose resistance displayed additive main effects, epistatic effects or both. This architecture has been frequently reported for other quantitative resistances (Young, 1996). Hence, in addition to main effect QTLs, significant epistatic interactions between QTLs have previously been reported in quantitative resistance against other fungus, such as *Phytophthora capsici* in pepper (Lefebvre and Palloix, 1996; Thabuis et al., 2003), *Rhizoctonia solani* in rice (Liu et al., 2014) or *Puccinia triticina* in wheat (Singh et al., 2014). However, genetic mapping studies considering epistatic interaction effects have not been performed so far in common bean. In this work, depending on the race and organ tested, the total phenotypic variation explained by main effect QTLs ranged from 2.64 to 23.89% (SAUDPC and SDC traits, respectively), whereas epistatic interactions explained a total phenotypic variation from 3.34 to 15.29% (PDC and LDC traits, respectively). Most of the epistatic interactions detected were due to loci without detectable QTL additive main effects, which show the importance of the epistatic effects in genetic resistance to anthracnose. Furthermore, resistant alleles came from the resistant parent PHA1037 more frequently, but they occasionally originated from the susceptible parent PMB0225, as observed in petiole resistance to race 1545. This result suggests that the susceptible parent also develops defense mechanisms, even though their activity could be insufficient to stop fungal progression.

The dissection of resistance into distinct phenotypic resistance components allows for a more precise QTL detection and facilitates the exhaustive selection of resistance factors in breeding programs. This type of approach has led to the identification of organ-specific defense mechanisms for resistance to *Phytophthora infestans* in potato (Gao et al., 2013) and *Colletotrichum graminicola* in maize (Balmer et al., 2013). This study has identified QTLs located in the same genomic region for resistance to different organs (*SDC*^*23*^-*4*, *SAUDPC*^*23*^-*4*, *LDC*^*23*^-*4.1*, *LAUDPC*^*23*^-*4*, *SDC*^*1545*^-*5*, *SAUDPC*^*1545*^-*5*, *PDC*^*1545*^-*5*, *PAUDPC*^*1545*^-*5*, *LDC*^*1545*^-*5*, *LAUDPC*^*1545*^-*5*, *SDC*^*1545*^-*8*, *SAUDPC*^*1545*^-*8*, *LDC*^*1545*^-*8*, and *LAUDPC*^*1545*^-*8*) or for different races of infection (*LDC*^*23*^-*3*, *SDC*^*1545*^-*3.1*, and *PAUDPC*^*1545*^-*3*), which are usually described as “generalist QTLs” (Lefebvre and Palloix, 1996; Thabuis et al., 2003). In addition, “specialist QTLs” have also been identified, which were involved in organ- or race-specific resistance (*SDC*^*23*^-*1*, *PDC*^*1545*^-*1*, *PAUDPC*^*1545*^-*1*, *LDC*^*23*^-*5.1*, *LAUDPC*^*23*^-*5.1*, *LDC*^*1545*^-*7*, *LAUDPC*^*1545*^-*7*, *LDC*^*23*^-*9* and *LAUDPC*^*23*^-*9*). However, it is not possible to conclude whether those genomic regions containing “generalist QTLs” resulted from the clustering of “specialist QTLs” or from the pleiotropic effect of a single gene. The presence gene clusters acting on the same trait is widespread among higher plants, and it has also been described in the common bean genome (Schmutz et al., 2014). Geffroy et al. (1999) pointed out that the origin of these clusters preceded the geographic separation of the wild common bean gene pools, as well as the role of selection in the emergence of such clusters, which might confer a selective advantage to the genotype that possesses them.

### Co-localization of QTL with resistance genes

The association between NL genes and QTLs conferring resistance to *Colletotrichum* species has been reported in several plant species. In maize, a gene conferring resistance to anthracnose stalk rot, caused by *C. graminicola*, encoded a CC-NB-LRR protein (Abad et al., 2006). The physical mapping of *RCT1*, a host resistance gene against *C. trifolii* in *M. truncatula* showed that *RCT1* was part of a complex locus containing numerous genes homologous to previously characterized TIR-NB-LRR resistance genes (Yang et al., 2007). In common bean, QTLs associated with anthracnose resistance were mapped in a cluster on the LG04, which was composed by CC-NB-LRR genes (Ferrier-Cana et al., 2003; Geffroy et al., 2009). Therefore, there is strong evidence that NB-LRR genes confer gene-for-gene resistance to *Colletotrichum* species in diverse plant hosts. Schmutz et al. (2014) stated that the majority of NL genes were physically organized in complex clusters in the common bean genome. In the present study, the homologous regions spanning 17 of the 26 main effect QTLs detected were positive for the presence of NL genes. The main effect QTLs detected on LG01 were co-localized with a cluster of 17 NL genes at the bottom of Chr1. The *Co-1* anthracnose resistance cluster is also located in this position of the Chr1, which includes *Co-1*, *Co-1*^*2*^, *Co-1*^*3*^ (Melotto and Kelly, 2000), *Co-1*^*4*^ (Gonçalves-Vidigal et al., 2011), *Co-1*^*5*^ (Gonçalves-Vidigal and Kelly, 2006), *Co-1*^*65-X*^, and *Co-1*^*73-X*^ (Campa et al., 2014). Regarding the linked genes *Co-w* and *Co-x* (Geffroy et al., 2008), Richard et al. (2014) have recently positioned *Co-x* at the end of Chr1 to a 58 kb region that comprises eight genes: three phosphoinositide-specific phospholipases C (PI-PlC), one zinc finger protein, and four kinases, which suggests that *Co-x* is not a classical NL gene. Moreover, genes for resistance to angular leaf spot, common bacterial blight, *Fusarium* root rot, and white mold have been located at the bottom of Chr1 (Miklas and Singh, 2007), as well as *Phg-1* and *Ur-9* genes, which confer resistance against *Pseudocercospora griseola* and *Uromyces appendiculatus*, respectively (Kelly and Vallejo, 2004; Gonçalves-Vidigal et al., 2011).

Resistance main effect QTLs to race 23 acting in stem and leaf organs were positioned in an important cluster of 41 NL genes on Chr4. Geffroy et al. (1999) stated that three specific resistant genes for anthracnose were clustered in this region, which originated either from the Mesoamerican BAT93 parent (*Co-9*) or the Andean Jalo-EEP558 parent (*Co-x*, *Co-y*). Furthermore, a major-effect QTL for resistance to isolate 45 (for leaf, stem, and petiole resistance) and a reverse-effect QTL (for leaf resistance) for resistance to isolate A7 (Geffroy et al., 2000), as well as the *Co-3* anthracnose resistance cluster (Ferreira et al., 2013; Campa et al., 2014) were also located in this genomic region of Chr4. On the other hand, the main effect QTLs detected at the top of LG08 were placed within a cluster of 17 NL, where the *Co-4* anthracnose resistance cluster is located (Melotto et al., 2004; Rodríguez-Suárez et al., 2007; Campa et al., 2014).

In addition, it is worth noting that the main effect QTLs detected on LG05 for resistance to race 1545 in stem, petiole and leaf were positioned within a 1.2 Mb region where the LRR gene Phvul.005G117900 is located. Based on genome sequence analysis, it can be considered an important candidate gene for the non-organ-specific QTL identified here. However, given that regions containing fast evolving genes, such NL genes, that are susceptible to chromosomal rearrangement and transposition or genomic duplication (Meyers et al., 2005), it is not possible to determine if the non-organ-specific resistance resulted from the pleiotropic effect of the Phvul.005G117900 gene or from the clustering of different genes, which could not be present in the reference genome sequence. Thereby, further studies on fine mapping of the target genomic regions would be necessary to draw definitive conclusions.

## Concluding remarks

The results stated herein provide essential information not only for a better understanding of the plant-pathogen interaction but also for the application of genomic assisted breeding for anthracnose resistance improvement in common bean. This research has also shown the importance of the epistatic effects in genetic resistance to anthracnose, which has never been studied so far. Thereby, both main and epistatic interaction effects of genes or QTLs should be considered for a successful application of MAS, which provides an opportunity to use a pyramiding strategy for durable resistance. As well as providing useful tools for MAS of anthracnose resistance in common bean, this work also offers valuable clues for further study on cloning the candidate gene corresponding to the non-organ-specific QTL for resistance to race 1545 located on Chr5.

### Conflict of interest statement

The authors declare that the research was conducted in the absence of any commercial or financial relationships that could be construed as a potential conflict of interest.

## Supplementary material

Figure S1 **Disease score distribution of the RIL population: (A) stem for race 23, (B) stem for race 1545, (C) leaf for race 23, (D) leaf for race 1545, and (E) petiole for race 1545**. Red line curve: normal probability distribution; blue arrows indicate the mean value of both parental lines in each environment.
