# Supplementary figures and images for: Uncovering the genetic architecture of Colletotrichum lindemuthianum resistance through QTL mapping and epistatic interaction analysis in common bean
Source: Front Plant Sci. 2015 Mar 17;6:141. doi: 10.3389/fpls.2015.00141 (PMC4362272; doi:10.3389/fpls.2015.00141)

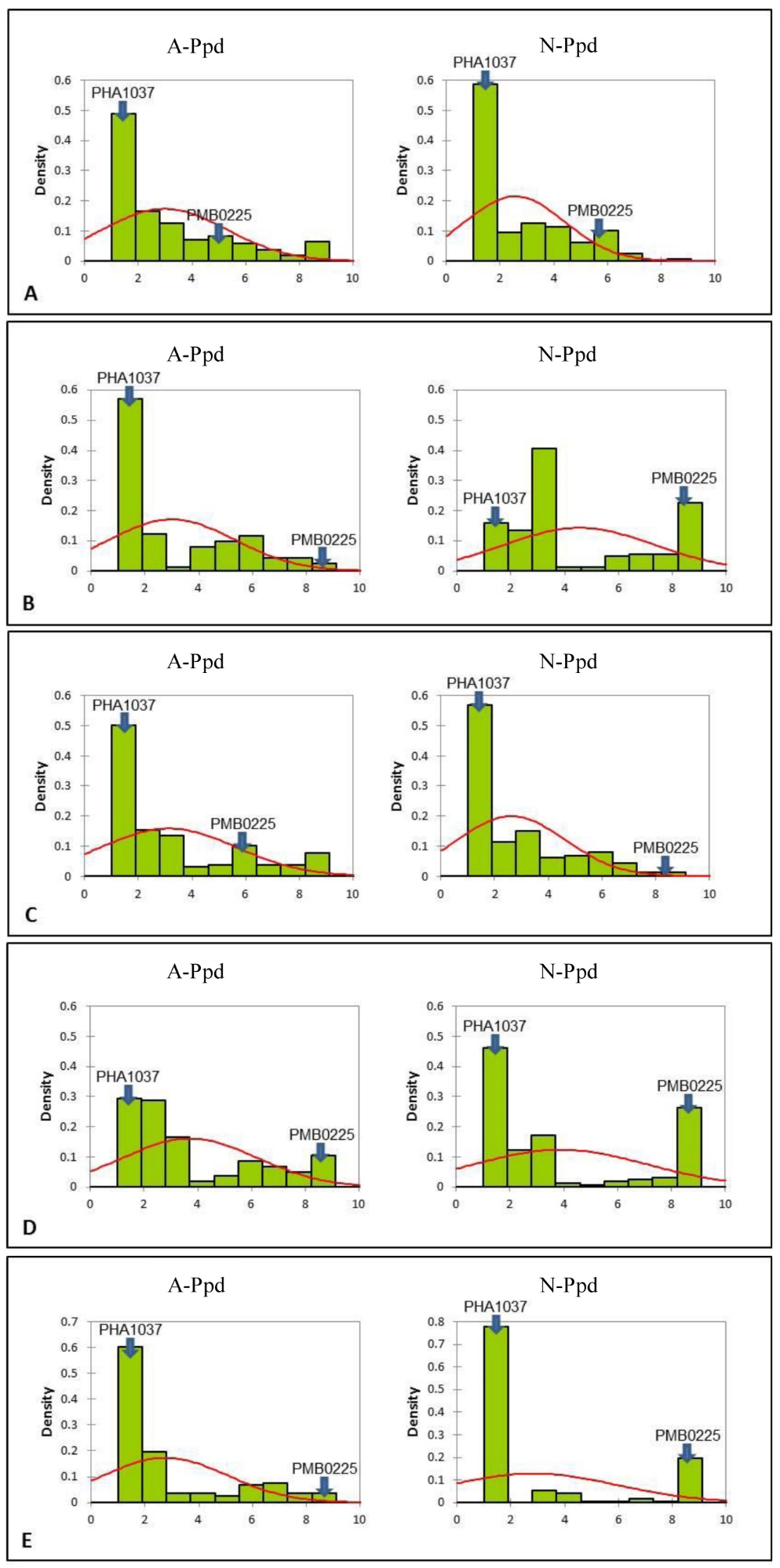

Supplement: Figure S1 — Disease score distribution of the RIL population: (A) stem for race 23, (B) stem for race 1545, (C) leaf for race 23, (D) leaf for race 1545, and (E) petiole for race 1545. Red line curve: normal probability distribution; blue arrows indicate the mean value of both parental lines in each environment. [file Image1.JPEG]
